# Filtration of Nanoparticle Agglomerates in Aqueous Colloidal Suspensions Exposed to an External Radio-Frequency Magnetic Field

**DOI:** 10.3390/nano11071737

**Published:** 2021-07-01

**Authors:** Maciej Marć, Andrzej Drzewiński, Wiktor W. Wolak, Lidia Najder-Kozdrowska, Mirosław R. Dudek

**Affiliations:** Institute of Physics, University of Zielona Góra, ul. Szafrana 4a, 65-069 Zielona Góra, Poland; m.marc@if.uz.zgora.pl (M.M.); w.wolak@if.uz.zgora.pl (W.W.W.); l.najder-kozdrowska@if.uz.zgora.pl (L.N.-K.); m.dudek@if.uz.zgora.pl (M.R.D.)

**Keywords:** bare and silica-coated nanoparticles, magnetic heating, filtration of nanoparticle agglomerates

## Abstract

The study investigated the phenomenon of the fast aggregation of single-domain magnetic iron oxide nanoparticles in stable aqueous colloidal suspensions due to the presence of a radio-frequency (RF) magnetic field. Single-domain nanoparticles have specific magnetic properties, especially the unique property of absorbing the energy of such a field and releasing it in the form of heat. The localized heating causes the colloid to become unstable, leading to faster agglomeration of nanoparticles and, consequently, to rapid sedimentation. It has been shown that the destabilization of a stable magnetic nanoparticle colloid by the RF magnetic field can be used for the controlled filtration of larger agglomerates of the colloid solution. Two particular cases of stable colloidal suspensions were considered: a suspension of the bare nanoparticles in an alkaline solution and the silica-stabilized nanoparticles in a neutral solution. The obtained results are important primarily for biomedical applications and wastewater treatment.

## 1. Introduction

In recent years, iron oxide nanoparticles and their hosting matrices have been subject to rapidly increasing development. This is due to their unique size-dependent magnetic properties, proven biocompatibility, the large chemical reactivity of their surface, and ease of synthesis [1,2,3,4]. In particular, magnetite (Fe${}_{3}$O${}_{4}$) and maghemite (γ-Fe${}_{2}$O${}_{3}$) are two main crystal phases, commonly used in various applications. The most common examples are magnetic resonance imaging [5,6,7], magnetic hyperthermia [8,9,10,11,12,13,14,15], drug delivery/release systems [16,17,18], immune response control [19,20], and environmental remediation [21,22]. There are several methods of preparing iron oxide nanoparticles [23,24,25,26,27,28,29,30], but their aqueous suspensions are often not stable and agglomerate quickly. The stability of the nanoparticle suspension can be achieved by various techniques, such as hydrothermal and solvothermal synthesis [31,32], or by applying a polymer layer or ionic layer [33,34,35,36]. In the case of ultra-small iron oxide nanoparticles (the term refers to the nanoparticles smaller than 50 nm [37]), the stability of the aqueous suspension can be achieved by an appropriately selected pH value of the aqueous solution. Upon contact with it, iron oxide nanoparticles acquire a pH-dependent surface charge: positive for pH < PZC, where PZC is the point of zero charge, and negative for the opposite case. The higher the electrostatic charge, the stronger the repulsion of nanoparticles opposing the agglomeration associated with the dipole–dipole magnetic interactions. The PZC values of magnetic nanoparticles vary depending on their preparation. In particular, the PZC of the magnetic Fe${}_{3}$O${}_{4}$ nanoparticles may range from 6.3 to 7.9 [38,39,40,41,42]. These very different PZC values result from different methods of synthesizing magnetic nanoparticles, different measurement methods, and the influence of the solution temperature. Generally, for biomedical applications, the nanoparticles with a hydrodynamic diameter smaller than about 30 nm and a narrow particle size distribution are required [43,44,45].

In this study, iron-oxide nanoparticles with an average size of about 10.5–11 nm (the PZC is about 6.6 [46]) were analyzed both as bare nanoparticles and the magnetic core of the silica-stabilized nanoparticles. The Fe${}_{3}$O${}_{4}$ surface coated in silica, due to the presence of surface silanol groups, can easily react with a variety of coupling agents to covalently attach specific ligands. In addition, silica stabilizes the iron-oxide nanoparticles by partially shielding the interaction of magnetic dipoles. Overall, such functionalization significantly increases the possibilities of using iron oxide nanoparticles, e.g., the silica coating allows them to survive in vivo [47,48]. It is worth noting that some authors have suggested that the addition of salt can significantly accelerate the sedimentation process of magnetite nanoparticles coated with silica in a magnetic field gradient [49]. In order to eliminate this effect, all nanoparticles were washed several times with distilled water.

In the neutral solution at pH = 7, silica-coated nanoparticles are negatively charged (their PZC is around pH = 2), which increases the electrostatic repulsion of nanoparticles, also hindering the formation of agglomerates. It is worth emphasizing that our recent publication showed [50] the possibility of controlling the electrostatic charge acquired by the silica surface when silica is filled with the magnetic nanoparticles heated by a RF magnetic field.

**Figure 1:**
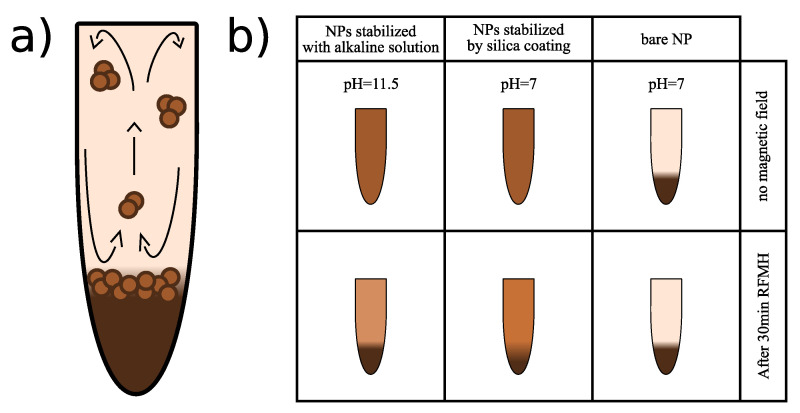
(**a**) Diagram of the circulation of magnetic nanoparticles in an aqueous solution subjected to a RF magnetic field. (**b**) The schematic drawings in the top row illustrate stable colloidal suspensions of bare nanoparticles at pH = 11.5, silica-stabilized nanoparticles at pH = 7, and the unstable suspension of bare nanoparticles at pH = 7. The lower schematic drawings show the sedimentation effect of magnetic nanoparticles as a result of exposure to an external RF magnetic field.

The main goal of this paper is to propose a fast, controllable, and economic method of separating nanoparticle aggregates from the (bare/coated) iron-oxide magnetic nanoparticles in an aqueous solution. On the other hand, the acquired results also showed that a RF magnetic field can cause undesirable effects of increased agglomeration that could be important in some applications, such as magnetic hyperthermia. These agglomeration effects are illustrated schematically in Figure 1.

The aggregation process driven by a RF magnetic field relies on localized thermal fluctuations induced by heated nanoparticles, which stimulate them to collide and stick together due to the magnetic dipole–dipole interactions. In addition, the presence of an external RF magnetic field causes the easy axes of the magnetic nanoparticles to align partially, oscillating with a small amplitude, which in turn enhances the strength of the magnetic interactions [51,52]. The key factor for the conducted research is that by using a properly selected pH value of the aqueous solution and amplitude of the magnetic field, it is possible to induce convective currents in some aqueous magnetic suspensions, which effectively filters out agglomerates of nanoparticles from the supernatant (except the smallest ones). This behavior occurs for the bare magnetic nanoparticles in an alkaline solution, and also for the silica-stabilized nanoparticles in the solution at pH = 7.

## 2. Materials and Methods

### 2.1. Chemicals

Iron(III) chloride hexahydrate and ammonium hydroxide, mesoporous silica nanoparticles, iron(II) sulfate heptahydrate, sodium hydroxide and hydrochloric acid were used. All reagents were used as received without further purification.

### 2.2. Synthesis

The main advantage of the precipitation process is that a large amount of nanoparticles can be synthesized. The applied method of co-precipitating nanoferrites from a precursor mixture of iron salts in ammonium hydroxide was derived from the technique proposed by Massart [53], providing magnetite nanoparticles with an average size of 10.5–11 nm.

The synthesis of the Fe${}_{3}$O${}_{4}$ nanoparticles was carried out according to the following procedure. The amount of 3.8 g of iron(II) sulfate heptahydrate FeSO${}_{4}$· 7H${}_{2}$O was dissolved in 100 mL of distilled water. Then the 7.2 g of iron(III) chloride hexahydrate FeCl${}_{3}$· 6H${}_{2}$O was dissolved in 100 mL of distilled water. Both iron salt solutions were mixed in one beaker and stirred at 400 rpm for 30 min. After this time, the rotational speed of the mechanical stirrer was increased to 1000 rpm. Then, 25 mL of a 25% ammonia solution was added dropwise at a rate of 1 drop/sec to the iron–salt solution while stirring it. The color of the solution then immediately turned black, indicating the formation of magnetite. The resulting suspension was mixed for another 30 min at a speed of 1000 rpm. Then, the appropriate amount of the suspension was taken and the washing procedure was carried out with a sodium base solution of a given pH value. The washing process consisted of separating the nanoparticles from the suspension with a neodymium magnet, collecting the supernatant from the top of the tube and supplementing the tube with the appropriate amount of sodium hydroxide solution, where the rinsing process was repeated five times. The obtained suspension was ready for testing or further processing in order to cover the nanoparticles with silica.

In order to obtain the silica-coated Fe${}_{3}$O${}_{4}$ nanoparticles, 10 mg of mesoporous silica nanoparticles was dissolved in 150 μL of 1 M sodium hydroxide [54], and the resulting solution was sonicated for 15 min at a temperature of 60 ${}^{\circ }$C. The previously obtained suspension of nanoparticles in an aqueous solution of sodium hydroxide was shaken and 20 mL was collected (approximately 120 mg of iron oxide). Then, 30 μL of dissolved silica was added dropwise (2 mg per 20 mL of suspension) and mixed for 40 min at a speed of 1000 rpm. The appropriate amount of 2 M HCl was added which made the resulting solution neutral (pH of about 7). Before testing, after further mixing for another 30 min, the resulting solution was also rinsed 3 times with distilled water, using a centrifuge at 18,000 rpm for 10 min.

### 2.3. Measurement Characterization

- Transmission Electron Microscopy. Conventional TEM images were obtained using the Fei Tecnai G2 F20 S Twin transmission electron microscope (Hillsboro, OR, USA).
- inViaTM Qontor® confocal Raman microscope using inVia Reflex Renishaw system equipped with Leica DM2700 and Leica N Plan EPI 50x/0.75na BD Objective. In this study, a laser with a maximum power of 50 mW was used. The following operating parameters were applied: 532 nm wavelength, 0.5% laser power, exposure time 10 s, measurement results averaged over the points of a 20×20 grid.
- XRD measurements using Malvern Panalytical’s Empyrean X-ray diffractometer was used. The operating parameters of the X-ray diffractometer were as follows: voltage 40 kV, current 40 mA, the wavelength of the CuKα-radiation 1.5406 Å.
- Dynamic Light Scattering, where particle hydrodynamic size measurements were carried out using Zetasizer Nano ZS analyzer (Malvern Instruments Ltd., Worcestershire, UK), operated in the 173${}^{\circ }$ backscattered mode on diluted aqueous solutions of nanoclusters.
- Radio-frequency magnetic field generator for heating a magnetic colloid suspension based on a copper coil with 6 turns. The inside of the coil was cooled by flowing water. The following parameters were used in the experiments: the frequency of the magnetic field f=100 kHz and the amplitude of the magnetic field $B_{0}=20$ mT.

## 3. Results

The preparation of a stable aqueous suspension of magnetic iron–oxide nanoparticles is usually the initial stage for functionalizing/stabilizing their surface according to the target application. In this case, the stability of the nanoparticle suspension was tested at different storage times and different values of pH ranging from 7 to 13 (iron-oxide nanoparticles dissolve in a strong acidic medium). Independently, the zeta potential was measured to verify the stability of the aqueous suspension of bare nanoparticles. It turned out that the zeta potential for a pH in the range of 11 to 12 is less than −40 mV, which ensures the stability of the suspension. By comparison, at pH = 7, the zeta potential is only about −16 mV.

**Figure 2:**
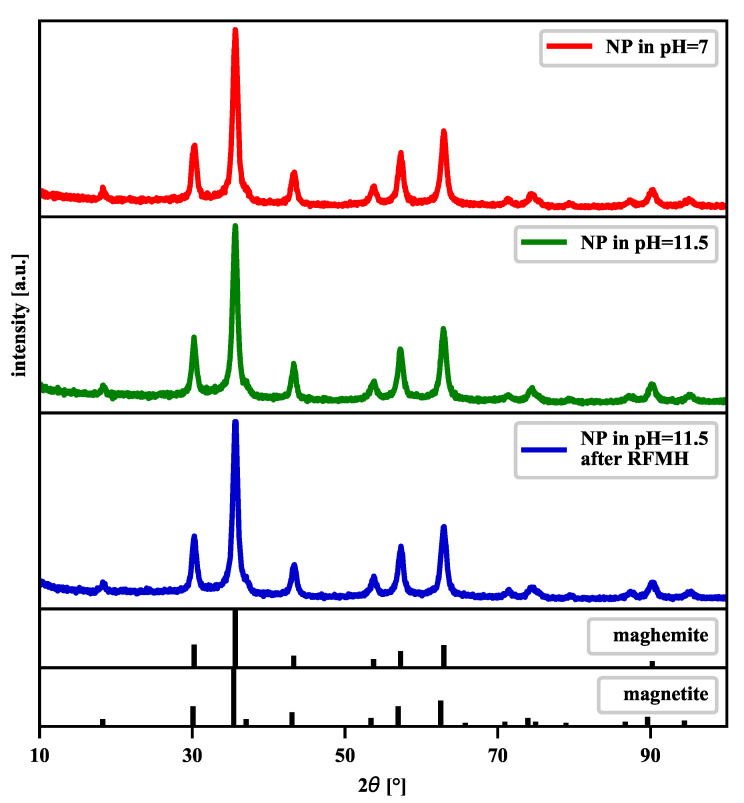
Wide-angle XRD patterns of the bare nanoparticles (NPs), NPs from the solution at the pH = 11.5 and NPs from the solution at the pH=11.5 subjected to the 1800-s RFMH. The X-ray diffractograms of the maghemite and magnetite powders are presented in the two narrow panels at the figure bottom [59].

In this study, the bare magnetic nanoparticles were prepared according to the chemical method described in Section 2. The nanoparticles have a spherical shape and a core-shell structure where the magnetic single-domain core has the Fe${}_{3}$O${}_{4}$ crystallographic structure, and the shell resembles a magnetically disordered material (see the discussion of this issue in the following papers [55,56], also by some of the current authors [18]). Under the conditions of direct contact with the environment, such small nanoparticles degrade over time in the γ-Fe${}_{2}$O${}_{3}$ structure. The X-ray diffraction (XRD) pattern of the samples with our magnetic nanoparticles in Figure 2 reveals that are dealing with both magnetite (Fe${}_{3}$O${}_{4}$) and maghemite (γ-Fe${}_{2}$O${}_{3}$), which is consistent with the fact that almost a month had passed from the production of these nanoparticles to the XRD measurements. It contributed significantly to the increase in the share of maghemite in relation to magnetite as a result of oxidation processes, but their relative proportion is difficult to determine because of their similar structures. Moreover, the method of estimating the percentage of magnetite and maghemite based on the deconvolution of two overlapping peaks, which worked very well for nanoparticles with an average size of 34 nm [57], would not be credible in our case due to wider peaks.

The mean nanoparticle size *D*, estimated from the most intense peak at $2\theta =35.6^{\circ }$ with the help of Scherrer’s equation [58], D=Kλ/βcos(θ), where *K* is the shape factor (0.9 here), λ is the wavelength of the X-rays (1.5406 Å here), β is the full-width at half-maximum (FWHM) of the peak (in radians) and θ is the Bragg angle, takes the value *D* that ranges from 10.5 nm to 11 nm (see Table 1).

Summing up, the XRD measurements show that exposure of the material to an aqueous solution of sodium hydroxide and radio-frequency magnetic heating (RFMH) does not significantly affect its phase composition.

**Figure 3:**
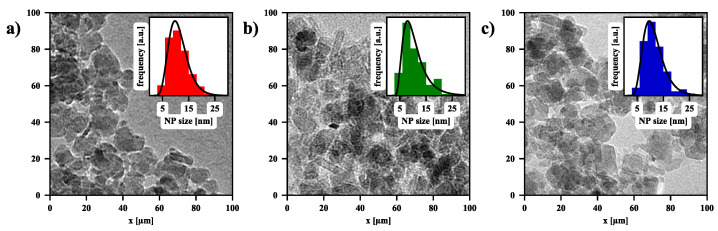
The TEM images for (**a**) the bare NPs, (**b**) NPs from the solution at the pH=11.5 and (**c**) NPs from the solution at the pH=11.5 subjected to the 1800-s RFMH (the particle size distribution histograms and log-normal fittings are presented as the insets).

The basic results concerning the morphology and size distribution of the nanoparticles [60] are shown in the transmission electron microscope (TEM) images in Figure 3a–c. These visualize nanoparticle assemblies from a dried magnetic colloidal suspension at three different environmental conditions: (a) bare iron oxide nanoparticles in an aqueous solution with a pH value of 7, (b) bare nanoparticles in an alkaline solution with a pH value of 11.5, and (c) bare nanoparticles in an alkaline solution with pH = 11.5 exposed to an external RF magnetic field of frequency f=100 kHz and amplitude $B_{0}=20$ mT. The TEM images show that in each case, the nanoparticles are polydisperse and their irregular geometry reflects roughly spherical shapes. Moreover, they indicate that the particle size distribution performs a log-normal function, which allows determining the average size of the particles of about 11±2 nm.

**Figure 4:**
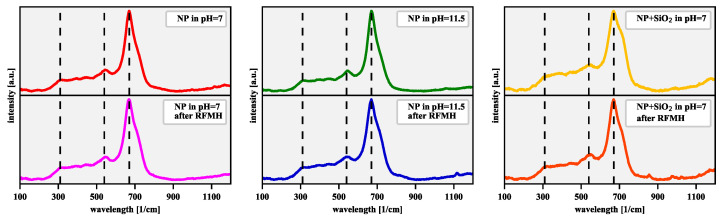
The Raman spectra with a substracted linear background before and after the 1800-s RFMH process for freshly prepared NPs: (left panel) bare in solution at pH=7, (middle panel) bare in solution at pH=11.5, and (right panel) silica-coated in solution at pH=7. The vertical dashed lines mark the positions of the Raman spectra peaks most characteristic of magnetite.

Raman scattered radiation measurements for which the reference spectra of iron oxidation products are known, carried out for freshly prepared (1–2 days old) nanoparticles, lead to the same conclusion. The magnetite is usually identified by its strongest peak at 670 cm${}^{-1}$, as others may be covered by peaks from its possible oxidation byproducts. A more reliable identification can be made when the following three peaks are visible: 310 (the $E_{g}$ mode), 540 (the $T_{2g}$ mode) and 670 (the $A_{1g}$ mode) cm${}^{-1}$, which was unambiguously determined in our measurements (Figure 4). The symbols in the brackets denote the irreducible representations of the optical phonon modes of the spinel structure [61]. The presence of the $A_{1g}$ mode is most often attributed to the symmetric stretching of oxygen atoms in the Fe${}_{3}$O${}_{4}$ tetrahedral group along the 〈111〉 direction, whereas the symmetric and asymmetric bending of oxygen with respect to iron in the tetrahedral environment are responsible for the $E_{g}$ and $T_{2g}$ modes [62,63]. Of course, also in this case, some oxidation of magnetite nanoparticles to maghemite ones cannot be excluded. Therefore, in the latter case, one can expect a slight shift in the positions of the peaks and their different relative intensities. Namely, for maghemite, the most intense $A_{1g}$ peak appears around 700 cm${}^{-1}$, and two broad peaks at 365 (the $T_{2g}$) and 511 cm${}^{-1}$ (the $E_{g}$ mode) are present [64,65].

The collected Raman measurements confirm the conclusion from the previous methods that the influence of the aqueous solution of sodium hydroxide and the RFMH process does not significantly affect the structure of the material. Moreover, the material responsible for the magnetic response of the freshly prepared nanoparticles is magnetite, but the maghemite content, as we have also verified using Raman spectra, increases significantly with time.

**Figure 5:**
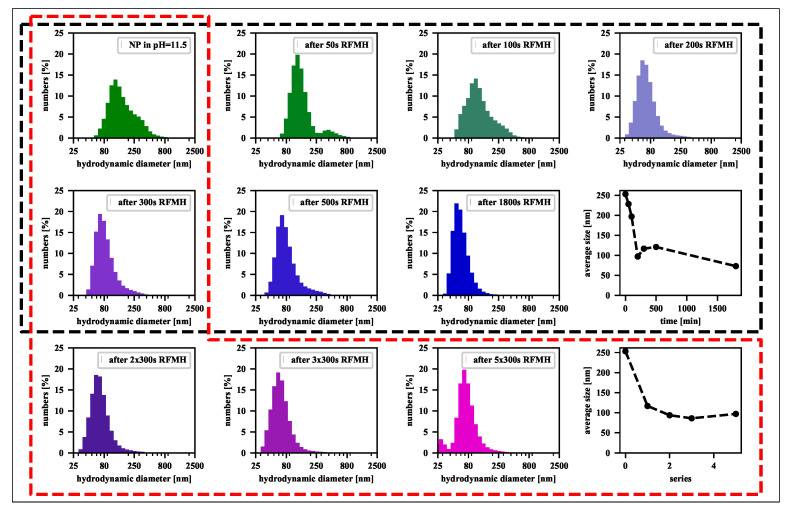
The histograms of the size distribution of NPs in the pH=11.5 solution for samples taken from the upper part of the Eppendorf tube: (the panels surrounded by a black dashed line) during the 1800-s RFMH process and (the panels surrounded by a red dashed line) during a series of 300 s processes, where a single process was applied 1, 2, 3 and 5 times. Each of the panel group is supplemented with the plot showing how the average particle size changes with time.

For direct insight into the agglomeration process caused by the RFMH, the dynamic light scattering (DLS) measurements on a series of samples representing a colloidal suspension of nanoparticles after various exposure times to a RF magnetic field (the two upper rows in Figure 5) were performed. In each case, the samples were taken from a solution at pH= 11.5 after the exposure time indicated in the legends. As one can see, in the first 200 s, the size distribution of aggregates changes quickly, but after 300 s, the maximum of the distribution stabilizes around 70 nm. Next, in practice, the maximum position remains stable at least until 900 s, which covers both the range of the convective circulation and upward ejections. Eventually, in the region where the fluctuations of the surface temperature vanish and its value stabilizes, the size distribution peak finally stops around 50 nm.

## 4. Discussion

Exposing the aqueous suspension of magnetic nanoparticles to an external RF magnetic field destabilizes the suspension (Figure 1). The energy of the applied RF magnetic field absorbed by the magnetic nanoparticles is transferred in the form of heat into the aqueous solution [66,67,68,69]. The resulting increased mobility of the magnetic nanoparticles significantly increases the number of collisions between them. Then, as a result of the magnetic dipole–dipole interactions, nanoparticles begin to aggregate and then fall under the influence of gravity. As an important source of heat, nanoparticles that have fallen to the bottom generate a significant temperature gradient between the lower and upper parts of the colloidal suspension. Since mainly small agglomerates remain in the upper part, the supernatant becomes highly transparent. Moreover, an external RF magnetic field causes the agglomeration processes to proceed very quickly, and the exposure time to the magnetic field is correlated with the amount of filtered larger agglomerates. The time of exposure of the colloidal suspension to the external RF magnetic field becomes a parameter to control the size of agglomerates remaining in the supernatant. In the experiment, the temperature of the upper layer of the colloidal suspension was measured by a pyrometer. When magnetic nanoparticles are the source of heat, due to the absorption of the energy of the alternating magnetic field, the temperature increase rate ΔT/Δt of the aqueous colloidal solution takes the following well-known form:

(1) $$\frac{\Delta T}{\Delta t}=\frac{PV}{m_{NP}C}\;$$

where *P* denotes the total volumetric heating power (in units of Wm${}^{-3}$), *C* is the mass weighted specific heat capacity [50,70], and *V* is the solution volume. Here, P=fA, where A=A(f) is the frequency dependent hysteresis loop area for an alternating magnetic field in terms of the nanoparticle magnetization *M*, and the external magnetic field $B=B_{0}\cos\left(2\pi ft\right)$. The value of *A* strongly depends on the size of the magnetic nanoparticles and there is always an optimal size of magnetic nanoparticles for which the heating rate is the highest [71,72]. This applies also to iron-based colloids in alkaline conditions [50].

**Figure 6:**
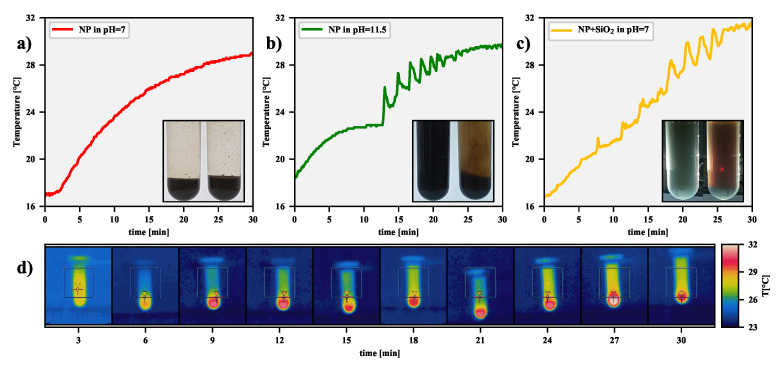
The plots in the top row show the surface temperature changes during the RFMH process for various NP suspensions: (**a**) bare NPs in solution at pH=7, (**b**) bare NPs in solution at pH=11.5, and (**c**) silica-coated NPs in solution at pH=7. The insets of the individual panels show two tubes with an untreated sample on the left and treated by the RFMH on the right. As the solution was the least transparent for the NPs + SiO${}_{2}$ suspension, both samples were additionally illuminated from behind. In this case, the red spot on the RFMH treated tube indicates that due to the large number of small agglomerates in the supernatant, short wavelengths of visible light are highly scattered. The bottom row (**d**) presents the time-lapse sequence of IR images for the NPs suspension at pH=11.5.

The effect of filtering out the magnetic agglomerates formed in the colloidal solution by means of a RF magnetic field can be confirmed by the dependence of ΔT on the time of exposure *t*. For the bare magnetic-nanoparticle dispersion in a solution at pH = 7, as can be seen in Figure 6a, the surface temperature increases monotonically with time until it becomes saturated, which is a well-known effect. On the contrary, for the bare magnetic nanoparticles in an alkaline solution at pH = 11.5, and also for the silica-stabilized nanoparticles in the solution at pH = 7, the plots in Figure 6b,c look distinctly different than in the previous case. For them, three different types of surface temperature behavior as a function of time can be observed during the RFMH process.

- An initially monotonic temperature increase, analogous to the temperature course in Figure 6a, corresponding to the heat transfer from nanoparticles to a fluid environment. Larger aggregates of nanoparticles begin to form and sink by gravity to the bottom of the tube (even the 50 s exposure time to an RF magnetic field is sufficient for noticeable particle segregation). Falling aggregates increase the temperature of the lower part of the magnetic suspension, activating a thermally driven convection circulation that carries finer aggregates.
- After about 10–12 min, rapid changes in the temperature of the surface colloid layer can be observed. During this time, the temperature difference between the bottom part and the upper part of the colloidal suspension reaches its maximum value, triggering the upward convective flow of liquid. For silica-stabilized magnetic nanoparticles, the effect is weaker.
- T(t) becomes saturated, which is accompanied by the disappearing circulation and smaller and smaller upward ejection of warm liquid. At this stage of the RFMH process, the distribution of nanoparticles in the colloid is practically unchanged.

The entire process is documented by the IR images in Figure 6d presenting the characteristic surface temperature evolution over time. In the first minutes, the temperature rises more or less evenly throughout the tube, but by the sixth minute, a hot spot is formed at the bottom, driving a convective circulation. At the ninth minute, one can see the convective upward flow begins, which at the tenth minute, quickly transforms into a violent ejection of warmed liquid. Subsequent IR images record the presence of the convective motion of liquid from the bottom layers upwards and then downwards.

It is worth noting, as shown in Figure 6, both for the suspension of bare nanoparticles in sodium hydroxide solution and for silica-stabilized nanoparticles in a neutral solution, the agglomeration and segregation processes follow the same scenario. In addition, the first 300 s turn out to be crucial for the filtration of aggregates from the solution. During this time, as already mentioned, there is a key change in the particle size distribution, and furthermore, the mean value of the particles decreases linearly, as shown in the last panel in the middle row of Figure 5. Moreover, all of this occurs even before the first rapid ejection of heated liquid towards the surface. Therefore, it was considered worthwhile to investigate an alternative procedure. Namely, after 300 s, the heating of the magnetic nanoparticle suspension was stopped, and the system was cooled down to the initial temperature. This step was then repeated several times to verify whether the cumulative effect of a series of short RFMH processes was more effective than one long RFMH process. The results of the measurements collected in Figure 5 prove that the efficiency of filtering out aggregates is similar for both approaches. Hence, a rapid upward ejection of the warm liquid is not essential, as it is a convective circulation that turns out to be crucial for the filtration process. Nevertheless, because when there is one long exposure to a RF magnetic field, there is no need to stop the process and cool the sample, this approach seems preferable from a practical point of view.

At the end of this section, an additional question was posed: how does the $B_{0}$ amplitude change affect the aggregation of single-domain magnetic nanoparticles by means of a RF magnetic field? As the reference system, the suspension of nanoparticles in an alkaline aqueous solution at pH = 11.5 was chosen.

**Figure 7:**
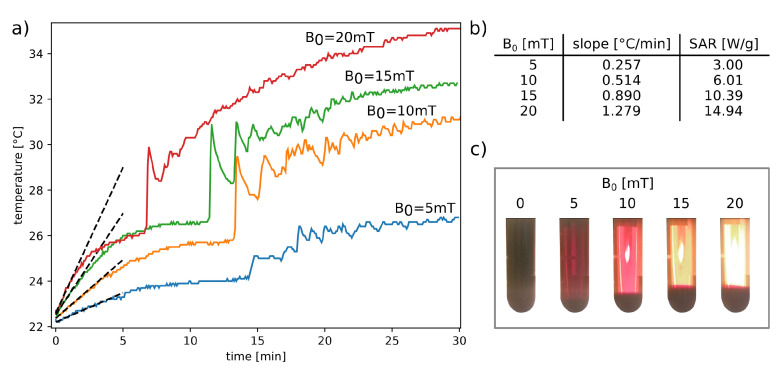
The left panel (**a**) presents the surface temperature changes during the RFMH process for various NP suspensions. Table (**b**) collects the specific absorption rates (SAR) of the nanoparticles for several amplitudes of the RF magnetic field [73], whereas the photo collage (**c**) shows how the supernatant changes after the 1800 s exposure to a RF magnetic field, depending on its magnitude. In the (**b**) case, the linear fit was determined from the first 60 s of the RFMH process. In the (**c**) case, each of the five tubes was additionally illuminated from the back with the same light source.

As Figure 7 shows, the nature of the phenomenon does not change qualitatively when an alternating magnetic field of a smaller amplitude is used. In any case, again, the same three different types of surface temperature behavior as a function of time can be observed during the RFMH process. Moreover, the higher the magnetic field intensity, the more transparent the supernatant, which shows that potentially, the amplitude of a RF magnetic field can play the role of another parameter controlling the particle aggregation process.

## 5. Conclusions

It was shown how an external RF magnetic field destabilizes a stable colloidal suspension of magnetic nanoparticles. On the one hand, the destabilization of the magnetic colloid by the appearance of large agglomerates, which are successively removed, can be used to prepare a colloid with very small agglomerates of nanoparticles, as presented in the paper. On the other hand, it was revealed that agglomeration processes in a RF magnetic field are inevitable and very fast. As a result, for example, the impact of hyperthermia will be much weaker in the case of the formation of agglomerates of nanoparticles in the colloidal suspension. Therefore, it is necessary to control the exposure time of the magnetic colloid to an alternating magnetic field. These issues are generally not discussed in the literature but they are very important for such applications as wastewater treatment [74] and various types of biomedical applications [75].

## Figures and Tables

**Table 1:** The values obtained from XRD analysis for individual samples.

	2θ [${}^{\circ }$]	β [rad]	*D* [nm]
NPs in pH = 7	35.63	0.0138	10.54
NPs in pH = 11.5	35.61	0.0137	10.84
NPs in pH = 11.5 after RFMH	35.63	0.0134	10.62
